# Pregnancy of unknown location

**DOI:** 10.6061/clinics/2019/e1111

**Published:** 2019-10-09

**Authors:** Pedro Paulo Pereira, Fábio Roberto Cabar, Úrsula Trovato Gomez, Rossana Pulcineli Vieira Francisco

**Affiliations:** Hospital das Clinicas HCFMUSP, Faculdade de Medicina, Universidade de Sao Paulo, Sao Paulo, SP, BR; Faculdade de Medicina FMUSP, Universidade de Sao Paulo, Sao Paulo, SP, BR

**Keywords:** Pregnancy, Ectopic, Progesterone, Human chorionic gonadotropin

## Abstract

Pregnancy of unknown location is a situation in which a positive pregnancy test occurs, but a transvaginal ultrasound does not show intrauterine or ectopic gestation. One great concern of pregnancy of unknown location is that they are cases of ectopic pregnancy whose diagnosis might be postponed. Transvaginal ultrasound is able to identify an ectopic pregnancy with a sensitivity ranging from 87% to 94% and a specificity ranging from 94% to 99%. A patient with pregnancy of unknown location should be followed up until an outcome is obtained. The only valid biomarkers with clinical application and validation are serum levels of the beta fraction of hCG and progesterone. A single serum dosage of hCG is used only to determine whether the value obtained is above or below the discriminatory zone, that means the value of serum hCG above which an intrauterine gestational sac should be visible on ultrasound. Serum progesterone levels are a satisfactory marker of pregnancy viability, but they are unable to predict the location of a pregnancy of unknown location: levels below 5 ng/mL are associated with nonviable gestations, whereas levels above 20 ng/mL are correlated with viable intrauterine pregnancies. Most cases are low risk and can be monitored by expectant management with transvaginal ultrasound and serial serum hCG levels, in addition to the serum progesterone levels. To minimize diagnostic error and intervene during progressive intrauterine gestation, protocol indicates active treatment only in situations when progressive intrauterine pregnancy is excluded and a high possibility of ectopic pregnancy exists.

## PREGNANCY OF UNKNOWN LOCATION: CLASSIFICATION AND FOLLOW-UP

Pregnancy of Unknown Location (PUL) is the term used to describe a situation in which a positive pregnancy test occurs, but a transvaginal ultrasound (TVUS) does not show intrauterine or ectopic gestation, nor does it show the retention of conception products (1). The incidence of PUL at centers specialized in the follow-up of early gestation varies from 8% to 10% (2,3) and fundamentally depends on the quality of the ultrasound examination performed, which in turn results from the examiner’s experience and the degree of resolution of the device used. The International Consensus of Ultrasound in Obstetrics and Gynecology determined that units specialized in early gestation should strive to maintain a PUL rate below 15% (4).

Ultrasonography is the best examination method for identifying the location of an early pregnancy. One study conducted in London at a unit specialized in early gestation showed that TVUS identified the location of the pregnancy in 91.3% of pregnant women. Of these women, 89.6% were diagnosed with intrauterine pregnancies (IUPs), 1.7% were diagnosed with ectopic pregnancies (EPs), and 8.7% were diagnosed with PUL (5). One great concern of PULs is that they are cases of ectopic pregnancy whose diagnosis might be postponed. TVUS is able to identify an EP with a sensitivity ranging from 87% to 94% and a specificity ranging from 94% to 99% when multiple exams are performed. With a single examination, TVUS identifies EPs with 73.9% sensitivity and 98.3% specificity (6). Regarding PULs, a common mistake is to perform TVUS alone. The adnexa might be located in a higher region, and only a pelvic abdominal ultrasound enables visualization and identification via a suggestive image to diagnose EP (7).

PUL rates and outcomes vary widely because of the different criteria used by several centers worldwide. Thus, experts from the United Kingdom, the United States, Belgium, the Netherlands, and Australia reached a consensus to standardize the ultrasound criteria for IUPs and EPs in 2011 (8). Faced with a positive pregnancy test, a woman can be classified into one of five categories based on her ultrasound findings:

1. Defined EP: extrauterine gestational sac with a yolk vesicle and/or embryo with or without cardiac activity
2. Probable EP: heterogeneous adnexal mass or gestational sac-like structure
3. PUL: absence of IUP or EP images
4. Probable IUP: presence of intrauterine echogenic gestational sac
5. Defined IUP: intrauterine gestational sac with yolk vesicle and/or embryo with or without cardiac activity

## CLASSIFICATION

A patient with PUL should be followed up until an outcome is obtained. The follow-up of a patient with PUL can result in four possibilities (8):

1. IUP: In this case, the ultrasonographic examination is performed early, and an intrauterine gestation is not identified. Where possible, the IUP is subdivided into viable IUPs and nonviable IUPs. Between 30% and 47% of patients with PUL are subsequently classified as IUP (1), where
  - Viable IUP denotes ultrasound signs that are compatible with gestational age
  - IUP of uncertain viability denotes definite ultrasound evidence of IUP; however, ultrasonographic signs are insufficient to indicate whether the gestation is viable
  - Nonviable IUP: ultrasound signs show anembryonic gestation, miscarriage, or the retention of the products of conception
2. Failed PUL (PULF): In this case, the spontaneous outcome of gestation occurs with negative human chorionic gonadotropin (hCG), but the exact location of gestation (i.e., whether intrauterine or ectopic) is never identified. Between 50% and 70% of PULs are classified as PULF. Thus, IUP and PULF represent forms of PUL considered low risk for complication (1)
3. EP: PUL should not be considered a synonym of EP or as EP until proven otherwise. Between 6% and 20% of PULs are classified as EPs (1)
4. Persistent PUL (PULP): Approximately 2% of patients with PUL are classified as PULP (9). In these cases, hCG does not decline spontaneously, an abnormal increase or plateau of hCG occurs (a variation of less than 15% in hCG titration over three consecutive 48-hour interval measurements), and TVUS does not show intrauterine or ectopic gestation. These cases are usually small EPs that are not visualized on ultrasound or represent the retention of the products of conception in the endometrial cavity with an active trophoblast. Cases of EP and PULP are considered high risk for complication (1). PULP is not a diagnosis, and its outcome depends on the following conduct (8):
  - EP not visualized: In this case, an increase in hCG titers occurs after uterine evacuation
  - Treated PULP: In this case, methotrexate (MTX) is administered without confirmation of the location of pregnancy via ultrasonography, laparoscopy, or uterine evacuation
  - Resolved PULP: This type is present when hCG titers are negative after expectant management or uterine evacuation, without evidence of chorionic villi on anatomopathological examination
  - Histological IUP: In this case, chorion villus is identified on anatomopathological examination after uterine evacuation

## FOLLOW-UP

PUL is not a diagnosis, and the patient should be followed up until a definitive diagnosis is made. Although consensus exists regarding the definitions and classification of PUL (8), unfortunately, globally accepted protocols are lacking for PUL follow-up assessments, which leads to stress and additional tests until an outcome is established. In a PUL follow-up, biomarkers are useful because they can help determine the location and viability of gestation. An ideal biomarker should be inexpensive, accurate, reproducible, and safe. Biomarkers can be classified into subgroups that reflect the function of the trophoblast, corpus luteum, endometrium, and angiogenesis (10). Most biomarkers are in the research phase and have not been tested in clinical trials. The only valid biomarkers with clinical application and validation are serum levels of the beta fraction of hCG and progesterone.

## hCG

hCG is the most widely used biomarker in PUL follow-ups. A single serum dosage of hCG cannot be used to predict the outcome of a PUL; rather, it is used only to determine whether the value obtained is above or below the discriminatory zone. The discriminatory zone or a discriminatory value indicates the value of serum hCG above which an intrauterine gestational sac should be visible on ultrasound (11). This value varies across various centers and fundamentally depends on the resolution degree of the ultrasound device, the examiner’s experience, and the hCG kit used by the laboratory. Currently, most services consider a discriminatory zone between 1,500 and 2,000/2,500 mIU/mL of hCG while using TVUS (12,13). When the hCG value is above the discriminatory zone and no intrauterine gestation is visible on TVUS, an EP should be suspected; however, it is possible to have a viable IUP even if the ultrasound does not show an IUP, and the hCG value is above the discriminatory zone. Several studies have documented the appearance of embryos with cardiac activity in the follow-up of pregnancies where the gestational sac was not visible on TVUS with hCG values above 2,000 mIU/mL (14-16). One study used a logistic regression model to calculate the probability of visualizing a gestational sac on TVUS by evaluating 651 pregnant women with vaginal bleeding and/or abdominal pain; of these cases, 366 were viable pregnancies. To obtain a 99% probability of visualizing an intrauterine gestational sac on TVUS, the discriminatory value of hCG should reach 3,510 mIU/mL. If hCG values of 1,500 and 2,000 mIU/mL are used, then the probabilities of visualizing a gestational sac are 80.4% and 91.2%, respectively (15). Other reasons for nonvisible gestational sacs with hCG values above the discriminatory zone are multiple gestation, the examiner’s experience, ultrasound resolution, obesity, uterine fibroids, uterine polyp, and the hCG standardization used by the laboratory (7,14,16).

To avoid the risk of a diagnostic error that might lead to the interruption of a viable pregnancy or a malformation due to MTX use (17,18), interventional conduct should not be instituted based on a single dose of hCG among patients with PUL who are hemodynamically stable. For a woman with PUL and hCG values above 2,000 mIU/mL, the most likely diagnosis is a nonviable IUP, which is twice as frequent as EP (14,19). On the other hand, EPs occur 19 times more frequently than viable IUPs if hCG titers are between 2,000 and 3,000 mIU/mL and are 70 times more frequent than viable IUPs if hCG titers are above 3,000 mIU/mL (14,20). Among PULs with hCG values between 2,000 and 3,000 mIU/mL, each viable IUP will be accompanied by 19 EPs and 38 nonviable IUPs. Therefore, the probability of a viable IUP in this situation is 1÷(1+19+38), which is approximately 2%. Likewise, if the hCG value is greater than 3,000 mIU/mL, each viable IUP will be accompanied by 70 EPs and 140 nonviable IUPs. In this situation, the probability of a viable IUP will be 1÷(1+70+140), which is approximately 0.5% (21).

The most commonly used method for the follow-up of a PUL is a serial serum hCG measurement. Kadar et al. (22) were the first to describe that the minimum increase in serum hCG in cases of progressive IUP over 48 hours was 66%. However, this study was performed with only 20 patients and a confidence interval (CI) of 85%. Subsequently, a study of 287 patients who presented with vaginal bleeding, abdominal pain, or both determined, with a 99% CI, that in 99% of the progressive IUPs with hCG less than 5,000 mIU/mL, the increase serum hCG over 48 hours was least 53% (23). More recently, a study of 1,249 patients with PUL showed, with a 99.9% CI, that the minimal increase in hCG in cases of progressive IUP over 48 hours was 35% (24). The increase in hCG at 48 hours in cases of progressive pregnancy differs depending on the initial concentration of this hormone: it increases 49% of the time when hCG is initially below 1,500 mIU/mL, 40% of the time when the initial hormone concentrations are between 1,500 and 3,000 mIU/mL, and 33% of the time when initial hCG is higher than 3,000 mIU/mL (25). Thus, a viable IUP can increase hCG more slowly than previously imagined and thereby decrease the risk of inadvertently interrupting a progressive gestation. The variation between the serum titers of hCG at 48 hours/hCG at 0 hours is called the hCG ratio, and it is the main method of follow-up with a case of PUL. An increase greater than 66% in an hCG titer at 48 hours (hCG ratio>1.66) is highly suggestive of a viable IUP (26). A decrease in hCG titers indicates a nonprogressive gestation, and an hCG ratio of <0.87 (hCG drop>13%) suggests a PULF with a sensitivity of 92.7% (95% CIs: 85.6%-96.5%) and a specificity of 96.7% (95% CIs: 90.0%-99.1%) (27). Conversely, if the hCG titers increase by ≤66% (hCG ratio≤1.66) or decrease by ≤13% (hCG ratio≥0.87), then the probabilities of EP and PULP increase (2).

Unfortunately, the pattern of variation in hCG titers over 48 hours cannot be used to diagnose the cases of PUL that progress to EPs with certainty. In cases of LG, hCG titers can increase, decrease, or stabilize. Most EPs exhibit a variation in hCG titers within 48 hours, which increase more slowly than viable IUPs or decrease more slowly than nonviable IUPs. Approximately 15% to 20% of EPs can increase hCG titers over 48 hours similar to a progressive IUP, whereas 10% of EPs show hCG behaviors at 48 hours similar to those of PULF cases (28). The sensitivity of the hCG ratio (hCG at 48 hours/hCG at 0 hours) to predict an EP ranged from 74% to 100%, and the specificity varied from 28% to 97% in a meta-analysis that analyzed the accuracy of serum hCG at the outcome of a PUL (29).

## PROGESTERONE

Serum progesterone (P) is a natural progestogen produced by the corpus luteum, placenta, and adrenal glands whose primary function is to maintain pregnancy. Single serum P doses have been used together with serum hCG doses in the follow-up of PULs. Serum P levels are a satisfactory marker of pregnancy viability, but they are unable to predict the location of a PUL. P levels below 5 ng/mL are associated with nonviable gestations, whereas levels above 20 ng/mL are correlated with viable IUPs. However, a considerable proportion of EPs present with P doses between 5 and 20 ng/mL, which limits its use in clinical practice to exclude the possibility of EPs (12). Serum P doses are useful in cases of PUL to identify patients with PULF and thereby minimize the examinations and days of follow-up because they are considered low risk, regardless of the location of the pregnancy. A prospective study evaluating 252 cases of PUL with P≤10 nmol/L found a positive predictive value for PULF of 98.2% (95% CIs: 96.8%-99.7%) (3).

## OTHER BIOMARKERS

Although only serum levels of hCG and P are used in clinical practice, numerous biomarkers have been studied as predictors of PUL such as creatine kinase, cancer antigen 125, inhibin A, inhibin pro-αC-related immunoreactivity, and insulin-like growth factor-binding protein (30,31). To date, only inhibin A doses have predicted PUL progression. Levels of inhibin A are significantly lower in cases of PUL that resolve spontaneously (i.e., PULF) compared with cases that result in EPs and IUPs. A study of 109 women with PULs demonstrated that all cases with titers of inhibin A ≤11 pmol/L resulted in PULF (31).

## MATHEMATICAL MODELS

To improve care for women with PULs, polynomial logistic regression models were developed to predict the outcomes of PULs as well as minimize the exams and follow-ups of patients classified as low or high risk. In this context, the M4 ([Bibr B32] [Bibr B33] 32-34) and M6 (35) models deserve mention. The M4 model was based on the hCG ratio (hCG at 48 hours/hCG at 0 hours) to calculate outcomes and classify patients. The insertion of the hCG values in the M4 model calculates the risks of PUL cases that result in EPs, IUPs, or PULF. This model classifies PULs as low risk if the estimated rate of EP is <5% and as high risk if the estimated rate of EP is ≥5%. Follow-up of low-risk cases is performed with a urine hCG test in 2 weeks (where the risk for a PULF is greater than the risk for an IUP) or TVUS within 1 week (where the risk for an IUP is greater than the risk for a PULF). For high-risk cases, the serum levels of hCG are measured and TVUS is performed every 48 hours. A multicenter study of 1,962 women with PUL showed that the M4 model identified 69.6% (95% CIs: 65.8%-73.1%) of the patients as low-risk PULs with a positive predictive value of 97.5% (95% CIs: 95.5%-98.6%). The same study reported a sensitivity of 88% (95% CIs: 79.9%-93.2%) regarding the diagnosis of high-risk PUL cases with outcomes of EPs or PULP (33). One study using this same model in the US population did not show the same accuracy, revealing a sensitivity of 49% in high-risk PUL cases. The sensitivity increased slightly to 55% after adjusting the diagnostic criteria of the American population to those criteria used for the British population (36).

Recently, a new polynomial logistic regression model called M6-P was developed, which involves two steps. Initially, a serum P dosage is performed. If the P titers are ≤2 nmol/L, then the patient is considered low risk, and the follow-up is performed via a urine hCG test in 2 weeks. In cases of P>2 nmol/L, the patient receives a second test that examines the hCG ratio (hCG at 48 hours/hCG at 0 hours). The M6-P model enables researchers to apply the serum P-value to the formula; if the patient uses P supplementation, then this test employs a variable to account for this use. Similar to the M4 model, if the EP risk is ≥5%, then the case is considered high risk, and the patient receives serum doses of hCG and TVUS every 48 hours. On the other hand, if the risk of EP is <5%, then a follow-up is performed via a urine hCG test in 2 weeks (when the risk for a PULF is greater than the risk for an IUP) or via TVUS within 1 week (when the risk for an IUP is greater than the risk for a PULF). The development of the M6-P model involved 2,753 cases of PUL and showed that the initial stage identified 16.6% of the cases as low risk, with only a single serum P-value; however, 2.9% of EPs were initially classified as low risk. With the application of the two steps, the M6-P model can identify 62.1% of all cases as low risk, with a negative predictive value of 98.6% and a sensitivity of 92%. A total of 7.9% of EPs were initially classified as low risk PULs. The possibility of erroneously classifying an EP as a low-risk PUL reinforces the need to explain the signs of possible complications to patients (35). The use of mathematical models to predict the outcome of a PUL and minimize the number of follow-up assessments and laboratory tests seems promising; however, to clinically validate these mathematical models, it remains necessary to test them in across multiple centers and different populations. These mathematical models are available at www.earlypregnancycare.com.

## SURGERY

A lack of visualization of the intrauterine gestational sac on TVUS, together with hCG values above the discriminatory zone, was considered an indication for laparoscopy. Currently, laparoscopy is reserved for cases of PUL with the symptoms or signs of hemoperitoneum (1).

Uterine evacuation via curettage (D&C) or manual vacuum aspiration (MVA) has been used primarily in the United States to differentiate EP from nonviable cases of IUP. Those who advocate for this conduct believe that the differentiation between EPs and nonviable IUPs is important in the future counseling of a new pregnancies because a history of EP increases the risk of recurrence in the event of a future gestation, and a nonviable IUP might be associated with recurrent miscarriage requiring further investigation. Another favorable point for uterine evacuation is that the unnecessary use of MTX would be avoided in up to 50% of cases (37). The presence of chorionic villi in the uterine evacuation material is used to diagnose nonviable IUPs. Up to 20% of the time, however, anatomopathological analyses do not show chorionic villi (38). One should then perform serum hCG tests immediately before uterine evacuation and 24 hours afterwards. If hCG titers decrease by at least 15% after uterine evacuation, then the diagnosis of a nonviable IUP will follow; on the other hand, if the serum levels of hCG stabilize or increase, then the most likely diagnosis will be EP, and the patient will be treated with MTX because the possibility of incomplete uterine evacuation is unlikely (39,40).

In cases of PUL, uterine evacuation should be an exception and only performed after the possibility of a viable IUP has been excluded. Unfortunately, the criteria commonly employed to exclude the possibility that viable IUPs might present a risk of viable gestation discontinuation (41).

## MTX

MTX has been used in PULP cases of clinically stable patients presumed to have EPs. Depending on patient preferences, MTX can be offered as an alternative to uterine evacuation. This drug is a chemotherapeutic agent antagonist of folic acid and shows a high success rate in selected EP cases (12). A 50-mg/m^2^ dose of MTX is injected intramuscularly, and if hCG does not decrease by at least 15% between days 4 and 7, then the same dose of MTX is repeated (42). Before initiating MTX treatment, it is essential that patients present with normal complete blood count, urea, creatinine, and liver enzyme tests. Side effects such as pneumonitis (43) and febrile leukopenia (44,45) are extremely rare at typical doses of MTX and have only been described as case reports in the medical literature. As a consequence of the teratogenic potential of this chemotherapeutic agent, the patient should wait at least 3 months before trying to become pregnant after MTX treatment (13,46). Like uterine evacuation, before administering MTX, one must ensure that a progressive IUP is not present. Unfortunately, diagnostic errors have been described, which lead to congenital malformations, abortions, and interruptions of desired pregnancies (17,18).

A prospective, multicenter study randomly selected 73 hemodynamically stable patients diagnosed with EP with hCG levels <1,500 mIU/mL or those diagnosed with PUL with hCG levels <2,000 mIU/mL who presented with plateau hCG levels (i.e., elevations or decreases in hCG titers between the day of diagnosis and 4 days after <50%). A total of 41 patients received MTX (1 mg/kg intramuscularly), and 32 women were considered pregnant. Success was considered when hCG decreased until it reached undetectable levels following the initial intervention. New interventions were not necessary for 76% of the MTX group or 59% of the expectant group (RR: 1.3, 95% CIs: 0.9-1.8). In the MTX group, nine patients required a new dose of MTX (22%); in the expectant group, MTX was administered to nine women (28%; RR: 0.8, 95% CIs: 0.4-1.7). In that study, MTX was not superior to expectant treatment in cases of EP or PUL with low hCG titers or plateau hCG titers (47).

Recently, cases classified as PUL and initially treated with MTX but who actually had hCG-producing tumors (i.e., gestational and nongestational choriocarcinomas) have been described (48,49). Although rare, this possibility should be kept in mind because the wrong diagnosis can postpone appropriate treatment and create resistance to the chemotherapeutic agent. Another possibility of diagnostic error is the presence of heterophilic antibodies. Individuals with heterophilic antibodies produce antibodies against antigens of other species (e.g., mice). Currently, immunoassay enzymes with mouse monoclonal antibodies are used in the serum dosage of hCG. Thus, positive tests do not indicate hCG production. This type of alteration has harmed several women who, because of positive serum hCG levels, were erroneously diagnosed with EP; in addition, those with hCG-producing neoplasia underwent laparoscopy, hysterectomy, or chemotherapy. Heterophilic antibodies are large molecules that are not present in the urine, so one should consider the possibility of heterophilic antibodies if the hCG test is positive in the blood but negative in the urine. However, urine serum levels can be negative because the small amount of hCG produces a test with low sensitivity. In these cases, it is necessary to use a heterophilic antibody blocker or to create diluted dosages of the sample for the correct diagnosis of the heterophilic antibody (50,51).

**Figure 1:**
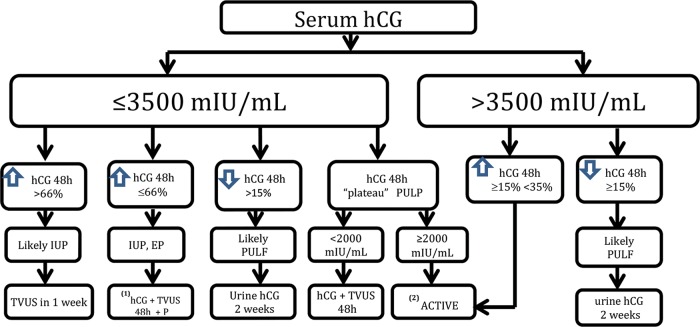
The algorithm employed by the Obstetric Clinic of the HCFMUSP in the follow-up of PUL cases. * Expectant: hCG up to a maximum of 3,500 mIU/mL (serum hCG+TVUS). **Active: MVA (hCG at 24 hours/hCG at 0 hours). Increase or decrease in hCG <15% 24 hours after MVA⇒MTX 50 mg/m^2^. Failure after one cycle of MTX, computed tomography, or magnetic resonance imaging of the chest, abdomen, and pelvis due to the possibility of chorionic gonadotropin-producing tumor.

Unfortunately, there is no consensus regarding the follow-up and timing of any intervention in cases of PUL. Our protocol is based on a discriminatory hCG value of 3,500 mIU/mL and variation in the hCG titers at 48 hours with the measurement of serum progesterone (Figure 1). Patients should be aware of the possibility of an EP and should quickly seek care in the event of its signs and symptoms. We choose expectant management for as long as possible, until the hCG level reaches the discriminatory value of 3,500 mIU/mL, and the patient is asymptomatic or oligosymptomatic. In the case of hCG titer plateau (i.e., variations of <15% across three consecutive doses within a 48-hour interval) with hCG values below 2,000 mIU/mL, we employ expectant protocol. On the other hand, we opt for active treatment when the plateau occurs with hCG titers >2,000 mIU/mL or hCG concentrations >3,500 mIU/mL, with an elevation of at least 15% over 48 hours. Currently, when we employ active treatment to avoid the unnecessary use of MTX, we prefer to perform MVA on an outpatient basis using local anesthesia via a paracervical block and aspiration with a 4-mm cannula. The material obtained via uterine evacuation is sent for anatomopathological study. If the hCG concentration does not drop by at least 15% after uterine evacuation, then the probable diagnosis will be EP, in which case, we prescribe intramuscular MTX at a dose of 50 mg/m^2^. In case of failure after the initial MTX cycle, we recommend the chest, abdomen, and pelvis examinations via computed tomography or magnetic resonance imaging to exclude the possibility of a hCG-producing tumor before starting a second cycle.

## CONCLUSION

Women with PULs should be followed up until a final diagnosis is established. In addition to TVUS, we routinely recommend abdominal pelvic ultrasonography for the complete visualization of the adnexa in cases of PUL. Fortunately, most cases are low risk and can be monitored by expectant management with TVUS and serial serum hCG levels, in addition to the serum P levels. However, the possibility of an EP, which has high morbidity and mortality rates during the first trimester of pregnancy, remains. In our view, one should have an active treatment to avoid delaying the EP diagnosis in certain situations. To minimize diagnostic error and intervene during progressive intrauterine gestation, our protocol indicates active treatment only in situations when progressive IUP is excluded and a high possibility of EP exists, i.e., when the hCG plateau is above 2,000 mIU/mL or the hCG concentration is above the discriminatory value of 3,500 mIU/mL with hCG titer elevation incompatible with progressive IUP.

Because consensus does not exist with regard to the follow-up of patients with PULs, we believe that additional studies are needed to elaborate algorithms to minimize laboratory exams and patient follow-ups, thereby reducing the costs and stress involved in the follow-up of these cases. Before being universally employed, mathematical models should be evaluated at several centers to ensure that they are tested with regard to different populations.

## AUTHOR CONTRIBUTIONS

Pereira PP contributed to the design of the revision and wrote the manuscript. Cabar FR wrote the manuscript. Gomez UT edited the manuscript and reviewed the final version of the manuscript. Francisco RPV coordinated the revision and edited and reviewed the final version of the manuscript.
